# Protein Phosphatase 1 (PP1) Is a Post-Translational Regulator of the Mammalian Circadian Clock

**DOI:** 10.1371/journal.pone.0021325

**Published:** 2011-06-21

**Authors:** Isabelle Schmutz, Sabrina Wendt, Anna Schnell, Achim Kramer, Isabelle M. Mansuy, Urs Albrecht

**Affiliations:** 1 Department of Biology, Unit of Biochemistry, University of Fribourg, Fribourg, Switzerland; 2 Laboratory of Chronobiology, Charité Universitätsmedizin Berlin, Berlin, Germany; 3 Brain Research Institute, Medical Faculty of University Zürich and Department of Biology of Swiss Federal Institute of Technology, Zürich, Switzerland; Vanderbilt University, United States of America

## Abstract

Circadian clocks coordinate the timing of important biological processes. Interconnected transcriptional and post-translational feedback loops based on a set of clock genes generate and maintain these rhythms with a period of about 24 hours. Many clock proteins undergo circadian cycles of post-translational modifications. Among these modifications, protein phosphorylation plays an important role in regulating activity, stability and intracellular localization of clock components. Several protein kinases were characterized as regulators of the circadian clock. However, the function of protein phosphatases, which balance phosphorylation events, in the mammalian clock mechanism is less well understood. Here, we identify protein phosphatase 1 (PP1) as regulator of period and light-induced resetting of the mammalian circadian clock. Down-regulation of PP1 activity in cells by RNA interference and *in vivo* by expression of a specific inhibitor in the brain of mice tended to lengthen circadian period. Moreover, reduction of PP1 activity in the brain altered light-mediated clock resetting behavior in mice, enhancing the phase shifts in either direction. At the molecular level, diminished PP1 activity increased nuclear accumulation of the clock component PER2 in neurons. Hence, PP1, may reduce PER2 phosphorylation thereby influencing nuclear localization of this protein. This may at least partially influence period and phase shifting properties of the mammalian circadian clock.

## Introduction

Many behavioral, physiological and metabolic functions are temporally organized and show daily rhythms, even in the absence of external timing cues. Temporal coordination of biological processes separates biochemically incompatible reactions, optimizes an organism's energy expenditure, and may improve overall performance. Circadian ( = ‘about a day’) oscillations are based on an autonomous clock mechanism that is phase-controlled and synchronized with the environment by external time cues, such as light or food. In mammals, the central circadian pacemaker is located in the suprachiasmatic nucleus (SCN), a structure in the ventral hypothalamus. Light, which serves as strong timing cue or Zeitgeber, directly impinges on the cellular oscillators of SCN neurons via the retinohypothalamic tract. The SCN can integrate this temporal information and coordinate all body clocks to establish and maintain adequate phase-relationships between different tissue clocks (reviewed in [1]).

The cell-autonomous clockwork that generates circadian oscillations is composed of interlocked transcriptional-posttranslational feedback loops [2]. These loops control the rhythmic expression of transcriptional activators and repressors whose interplay generates rhythms of approximately 24 hours. In the essential core feedback loop of the mammalian clock, two transcriptional activators, BMAL1 [3], [4] and CLOCK (or NPAS2 in the brain) [5], [6], [7], drive the expression of the *Period* (*Per1-3*) [8], [9] and *Cryptochrome (Cry1-2)* [10] genes via binding to E-boxes on the promoters of these genes. PER and CRY proteins form complexes and re-enter the nucleus, where they inhibit their own transcription [11], [12], [13]. This core loop can directly be influenced by environmental resetting signals via cAMP response element binding protein (CREB)-mediated induction of *Per* gene expression [14], [15]. This induction of the *Per* genes is involved in the light-mediated readjustment of the mammalian circadian oscillator to environmental time [16], [17], [18], [19].

Post-translational modifications of several clock components are required to adjust the period length of the feedback loops to 24 hours and to allow proper phase adjustments of the clock. Among these modifications, protein phosphorylation plays a critical role. Most clock proteins can be phosphorylated at multiple sites to determine their stability, activity and subcellular localization (reviewed in [20], [21]). The clock component PER2 appears to be highly regulated by phosphorylation. PER2 has at least 21 phosphorylated residues [22] and several kinases were described as regulators of PER2 [23], [24], [25]. A mutation found in the human *Per2* gene causes familial advanced sleep phase syndrome (FASPS) [26]. Interestingly, mutation of the FASPS site (S662) of an hPER2 transgene affects the period of the circadian oscillator in mice in opposite directions depending on whether the mutation mimics hypo- or hyperphosphorylation [27]. This suggests that the balance of protein kinase and phosphatase activity that controls the net phosphorylation state of every phosphorylated substrate is critical to allow optimal clock functioning. Protein serine/threonine phosphatases have been implicated in the regulation of circadian rhythms in dinoflagellates [28], neurospora [29] and the fruit fly [30], [31]. In mammals, phosphatases were shown to affect the expression of the clock component PER2 in cell culture [32], [33], indicating evolutionary conserved mechanisms. However, their precise function in the mammalian circadian mechanism has not been investigated *in vivo*.

Here, we examined the impact of protein phosphatase 1 (PP1) on circadian rhythmicity and photic entrainment of the mammalian circadian clock. PP1 is one of the major and most abundant eukaryotic protein serine/threonine phosphatases and has been implicated in various biological processes such as cell division, metabolism, synaptic plasticity, transcription and translation (reviewed in [34]). Activity, substrate specificity and intracellular localization of this enzyme are tightly regulated by the interaction with over 50 established or putative targeting and regulatory subunits [34], [35]. To study the function of PP1, PP1 activity was modulated in cells and in the brain of transgenic mice by inducible expression of the regulatory protein inhibitor-1 (I-1) or Nuclear Inhibitor of PP1 (NIPP1). Our results indicate that PP1 influences the period length of the mammalian circadian oscillator and is involved in mediating the response of the oscillator to entraining light information. At the cellular level, PP1 appears to be implicated in the regulation of the subcellular localization of the PER2 protein. Hence, PP1 is an important opponent of kinases within the mammalian circadian clock.

## Results

### Protein phosphatase 1 (PP1) influences circadian period length *in vitro*

**Figure 1:**
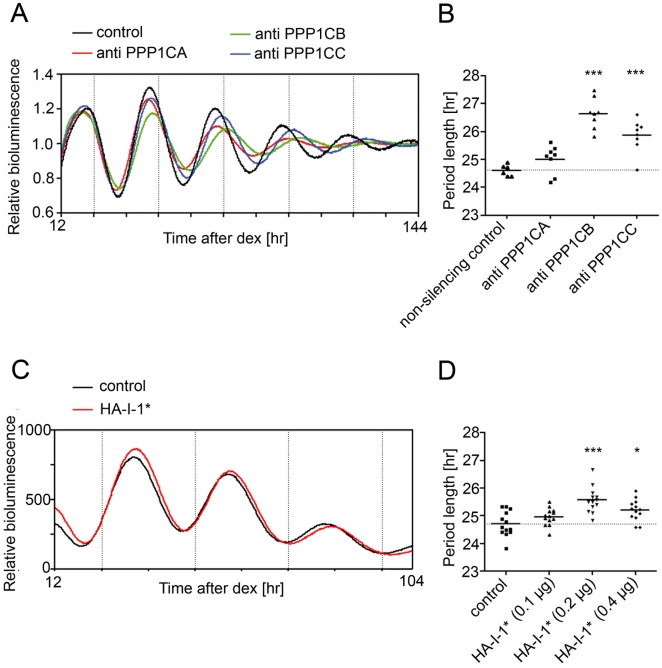
Reduction of PP1 activity increases period length *in vitro*. (A) Representative real-time bioluminescence records of U-2 OS cells harboring a *Bmal1* luciferase reporter vector that were lentivirally transduced with a construct containing scrambled RNAi (non-silencing control) or RNAi constructs against the catalytic subunits of PP1 (PPP1CA, PPP1CB, and PPP1CC; representative experiment out of three independent experiments; mean of three cultures). (B) Average period length of *Bmal1 luc* oscillations in U-2 OS cells lentivirally transduced with RNAi constructs against the catalytic subunits of PP1. Period length was determined in three independent experiments (with n = 2–3 cultures). (C) NIH 3T3 cells were transfected with a *Bmal1 luciferase* reporter vector together with either the empty expression vector or an expression vector for I-1* (0.2 µg). Cells were synchronized with dexamethasone, and luciferase activity was measured in real-time using a LumiCycle apparatus. Luciferase counts are plotted as mean of 3 cultures (representative experiment out of four independent experiments). (D) Average period length of *Bmal1* oscillations of transfected NIH 3T3 cells. Cells were transfected with 1.5 µg luciferase reporter vector, 0.1 µg pCMV-SEAP and 0.1 µg, 0.2 µg or 0.4 µg of empty expression vector/HA-I-1* expression vector (n = 13 individual cultures). *** p<0.001, * p<0.05 indicating significance (1-way Anova).

To get insights in the function of PP1 in the mammalian clock mechanism, we reduced the expression of various catalytic subunits of PP1 in human osteosarcoma U-2 OS cells by RNA interference. These cells contain a *Bmal1* promoter *luciferase* reporter that is stably integrated into the genome allowing the monitoring of circadian rhythms *in vitro* [24]. In mammals, three genes encode the PP1 catalytic subunits PPP1CA, PPP1CB, and PPP1CC [34]. These subunits are ubiquitously expressed and are targeted to varying but distinct intracellular locations by the interaction with regulatory proteins. The expression of PPP1CA, PPP1CB or PPP1CC was reduced by lentiviral infection of U-2 OS reporter cells with RNAi constructs specific for the respective subunits with more than 75% knock-down efficiency at the mRNA level (Figure S1A). Cells were synchronized with dexamethasone and the rhythmic bioluminescence was continuously monitored for several days. As a result, knock-down of PPP1CA, PPP1CB or PPP1CC provoked lengthening of the circadian period of U-2 OS cells of up to two hours (Figure 1A, 1B, S1B).

In a second approach we wanted to interfere with PP1 activity in a biochemical manner. We used NIH 3T3 mouse fibroblasts that express a constitutively active form of inhibitor-1 (I-1), an endogenous inhibitor of PP1 [36]. The constitutively active form corresponds to a short form of I-1 (amino acids 9 to 54), in which the threonine residue that is normally phosphorylated by protein kinase A is substituted by a phosphomimicking aspartic acid residue [37]. This constitutively active form of I-1 is designated I-1*. NIH 3T3 cells were transfected with a *Bmal1 luciferase* reporter together with either empty expression vector or increasing amounts of an expression vector for HA-tagged I-1*. Similar to the knock-down experiments, the expression of HA-I-1* lengthened the period of the *Bmal1 luc* oscillations (Figure 1C). This lengthening was dose-dependent for two concentrations of HA-I-1* expression vector (0.1 µg and 0.2 µg) and appeared to reach saturation at a concentration of 0.4 µg (Figure 1D). I-1* expression led to periods that were up to 50 minutes longer than the period of control cells. Altogether, these results indicate that PP1 is involved in the determination of the circadian period length in mammalian cells.

### I-1* mutant mice tend to have a longer free-running period

To assess the importance of PP1 on circadian behavior, PP1 activity was reversibly reduced specifically in the brain of transgenic mice (and not in other tissues). Mutant mice expressed inducibly the constitutively active form of inhibitor 1 (I-1*) [38]. Expression of I-1* was regulated by a tetracycline-controlled transactivator (rtTA2 transgene) system under the control of the Ca^2+^/calmodulin-dependent protein kinase alpha (*CaMKIIalpha*) promoter [39] that is also active in the SCN [40]. Double transgenic mice (I-1* mutant mice; rtTA2^+^/I-1*^+^) were treated with doxycycline in order to induce I-1* mRNA expression in the forebrain and hence leading to a reduction of PP1 activity. Control mice were animals having either one of the transgenes or no transgene (rtTA2^−^/I-1*^−^; rtTA2^−^/I-1*^+^; rtTA2^+^/I-1*^−^). They underwent the same treatment as indicated for double transgenic mice.

**Figure 2:**
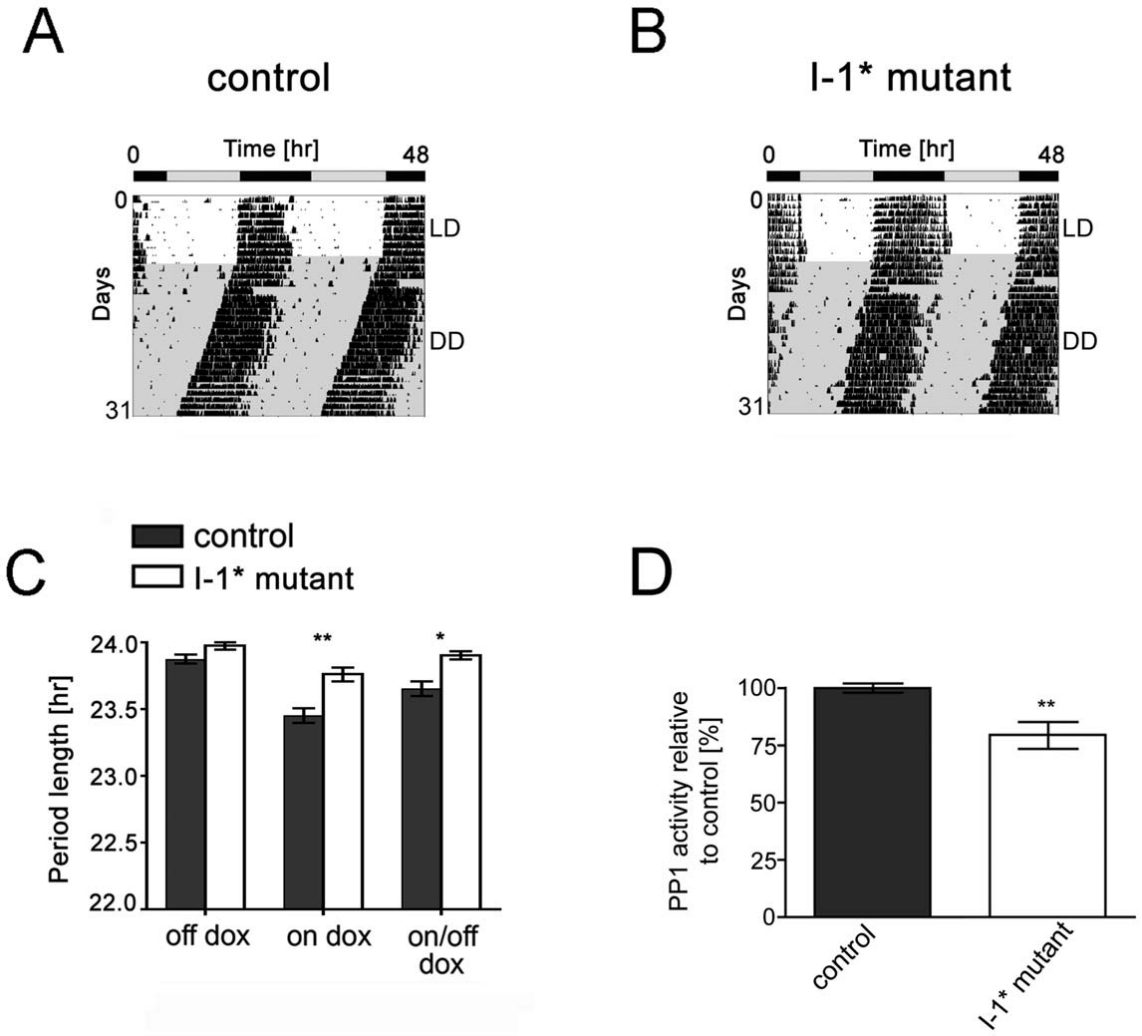
Dox-dependent and reversible lengthening of the circadian period of I-1* mutant animals. (A, B) Representative locomotor activity records of control (A) and I-1* mutant mice (B) under doxycycline treatment. Wheel-running activity is shown under free-running conditions in constant darkness (DD). (C) Average period length of control and I-1* mutant mice as determined by χ^2^-periodogram analysis under off dox, on dox and on/off dox conditions. Data are represented as mean ± SEM: control (off dox 23.88±0.03 h, n = 14; on dox 23.45±0.06 h, n = 14; on/off dox 23.65±0.05 h, n = 14), I-1* mutant (off dox 23.98±0.03 h, n = 9; on dox 23.76±0.05 h, n = 9; on/off dox 23.90±0.03 h, n = 9); ** p<0.01, * p<0.05 indicating significance (2-way Anova with Bonferroni post-test). (D) PP1 activity (% total activity) was measured in extracts from SCN tissue in control and I-1* mutant mice under doxycycline treatment. PP1 activity of control animals was set to 100% and mutant values were calculated in relation. Represented is the mean ± SEM (n = 8, ** p<0.01 indicating significance (unpaired t-test)).

We analyzed the wheel running locomotor activity before the doxycycline treatment (off dox), during the doxycycline treatment (on dox) and after the doxycycline treatment (on/off dox). Control and I-1* mutant mice entrained similarly to a 12-h light/12-h darkness cycle (LD 12∶12 h cycle) under on dox conditions (Figure 2A, 2B) and displayed stable free-running activity rhythms when maintained in constant darkness (DD; Figure 2A, 2B). Similar to the lengthening of the period in cells expressing I-1*, I-1* expression in the brain appears to provoke a longer free-running period compared to control mice (Figure 2C, on dox). This phenotype was not present under uninduced conditions (mutant off dox). Of note is the fact that doxycycline appears to shorten the period length in all genotypes as revealed by repeated measures 2-way ANOVA and paired t-test analysis (Table S2). The genotype as well as the treatment affected the results (F = 17.73, DFn = 1, DFd = 42, p = 0.0004; F = 32.74, DFn = 2, DFd = 42, p<0.0001, respectively) and the matching was effective (F = 2.65, DFn = 21, DFd = 42, p = 0.0035). However, interaction between genotype and treatment appears to be significant (F = 3.79, DFn = 2, DFd = 42, p = 0.03) indicating that the p values are difficult to interpret since the shortening effect of doxycycline is different depending on the genotype. In contrast to cells expressing I-1*, we observed only a trend towards a longer period in mice expressing I-1*. This is probably due to a less efficient reduction of PP1 activity in our mice compared to the knock-down in cells. To confirm I-1* expression and consequent reduction of PP1 activity in the SCN, we measured PP1 activity in the SCN of control and I-1* mutant mice under on dox conditions. PP1 activity was significantly reduced by 20% in I-1* mutant mice compared to control animals (Figure 2D).

### Protein phosphatase 1 is involved in light-induced clock resetting

**Figure 3:**
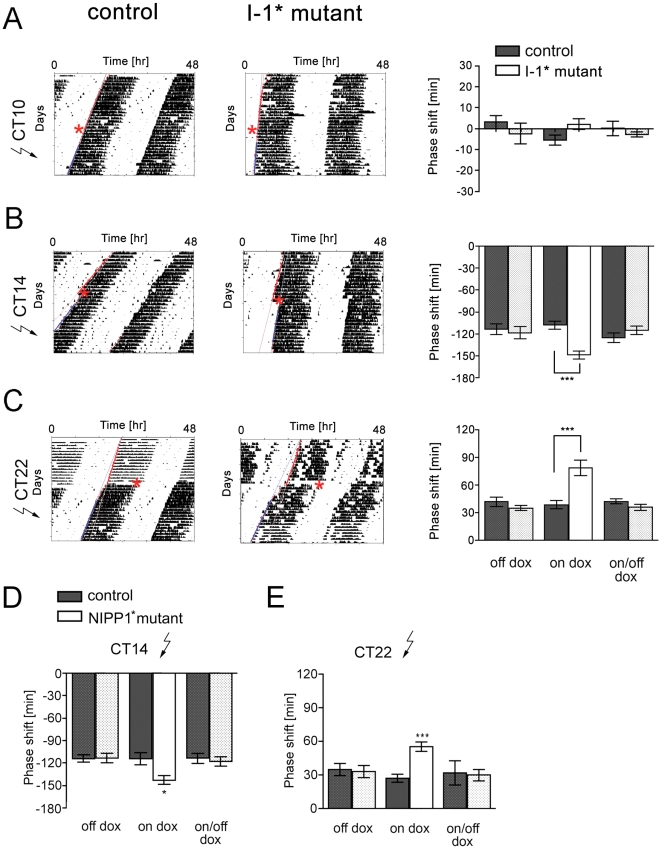
Light-induced resetting is altered in I-1* and NIPP1* mutant mice. (A–C) Shown are representative locomotor activity records before and after a 15-min light pulse of control (left panels) and I-1* mutant mice (middle panels) under on dox conditions. Red asterisks indicate the timing of the light pulse. The light pulse was administered at CT10 (A) (off dox n = 8 control, n = 3 mutant; on dox n = 20 control, n = 12 mutant; on/off dox n = 8 control, n = 3 mutant), CT14 (B) (off dox n = 11 control, n = 9 mutant; on dox n = 21 control, n = 15 mutant; on/off dox n = 12 control, n = 8 mutant) or CT22 (C) (off dox n = 14 control, n = 11 mutant; on dox n = 19 control, n = 13 mutant; on/off dox n = 15 control, n = 9 mutant). Red lines represent the onset of activity before and blue lines the onset of activity after the light pulse, respectively. The quantification of the phase shifts under off dox, on dox and on/off dox conditions is indicated in the right panels. (D–E) Light-induced phase shifts in control and NIPP1* mice after a light pulse at CT14 (D) (off dox n = 6 control, n = 3 mutant; on dox n = 9 control, n = 8 mutant; on/off dox n = 3 control, n = 5 mutant) or CT22 (E) (off dox n = 6 control, n = 3 mutant; on dox n = 8 control, n = 8 mutant; on/off dox n = 3 control, n = 5 mutant) under off dox, on dox, and on/off dox conditions. Significant differences were determined by 1-way Anova; *** p<0.001; * p<0.05. Presented is the mean ± SEM.

In addition to the importance of phosphorylation events for steady state oscillator functions, protein phosphorylation is an important mediator of light-induced resetting of the clock (for review see [41]). Thus, we analyzed the ability of I-1* mutant mice to reset the clock in response to light presented during the subjective night. We performed light pulses using an Aschoff type I protocol [42] under induced (on dox) and uninduced conditions (off dox and on/off dox). Animals were maintained in constant darkness and 15-min light pulses of 400 lx were administered. The light pulses corresponded to a pulse at circadian time (CT) 10 which is during the subjective day and should not have an effect on the oscillator, and light pulses at CT14 and CT22, which should provoke a phase delay and phase advance of the oscillator, respectively. Both, control and I-1* mutant mice, did not phase shift their locomotor behavior in response to light presented at CT10 under uninduced or induced conditions (Figure 3A). Interestingly after a light pulse administered at CT14, I-1* mutant mice displayed phase delays of −148.8±5.5 min (mean ± SEM) that were approximately 40 min greater than the phase delays of control animals (−107.9±5.5 min; Figure 3B). This phenotype was due to I-1* expression and reduction of PP1 activity as it was fully reversed by suppressing transgene expression (on/off dox condition; Figure 3B). Similar to the more pronounced phase delays, I-1* mutant mice also showed larger phase advances compared to control mice after a light pulse presented at late subjective night (CT22; 78.46±8.4 min for I-1* mutant and 38.34±4.45 for controls, respectively; Figure 3C). Again, phase advances were similar in control and I-1* mutant mice before doxycycline induction (off dox) or when transgene expression was suppressed (on/off dox) indicating that PP1 activity is involved in the observed resetting phenotypes.

The action of PP1 in determining the period length of the mammalian clock and in modulating the response of the oscillator towards light may be via dephosphorylation events in the cytoplasm, in the nucleus, or both. To investigate the role of PP1 specifically in the nucleus, we measured circadian parameters in mice expressing a fragment of the Nuclear Inhibitor of PP1 (NIPP1). This fragment contains the PP1 inhibition domain and the nuclear localization domain of NIPP1 and is constitutively active in the nucleus of cells (NIPP1*; [43]). NIPP1* was expressed in an inducible manner using the doxycycline-dependent rtTA2 and the *CaMKIIalpha* promoter in a similar manner as in the I-1* animals. NIPP1* mice had robust free-running rhythms in DD (Figure S2A, S2B) with a trend towards a slightly longer period under on dox conditions (Figure S2C). Similar to I-1* mutant mice, NIPP1* mutants showed significantly larger phase delays (Figure 3D, on dox) and phase advances (Figure 3E, on dox) after light pulses at CT14 and CT22, and this could be fully reversed after dox treatment (Figure 3D, 3E, on/off dox).

Altogether, these results suggest that PP1 is involved in the mechanism that mediates resetting of the clock in the brain in response to the Zeitgeber light. Our results further indicate that this process is most probably influenced by nuclear PP1 activity.

### PP1 affects PER1 and PER2 accumulation

Light-induced resetting of the circadian clock involves activation of several signaling cascades and phospho-CREB mediated induction of the *Per* genes in the SCN [14], [15], [44], [45]. This light-dependent induction of the *Per* genes (*Per1* and *Per2*) is suggested to affect the circadian oscillator in the SCN in response to light and consequently to phase-adjust the oscillator [46].

To test whether PP1 impinges on *Per* gene induction, we measured light-mediated *Per1* and *Per2* induction in the SCN in control and I-1* mutant mice at the beginning or the end of the subjective night. Interestingly, we observed a similar induction of *Per1* and *Per2* one hour after a light pulse at CT14 (Figure S3A, S3B) or CT22 (Figure S3C, S3D) in both I-1* and control mice. Quantifications and representative micrographs do not reveal any differences in the magnitude or localization of gene induction within the SCN between control and mutant mice. Thus, PP1 activity in the brain is probably less important for the induction of *Per* expression in the SCN in response to light. Consequently, PP1 may act on the phase shifting capacity of the circadian oscillator via another, maybe post-transcriptional mechanism.

**Figure 4:**
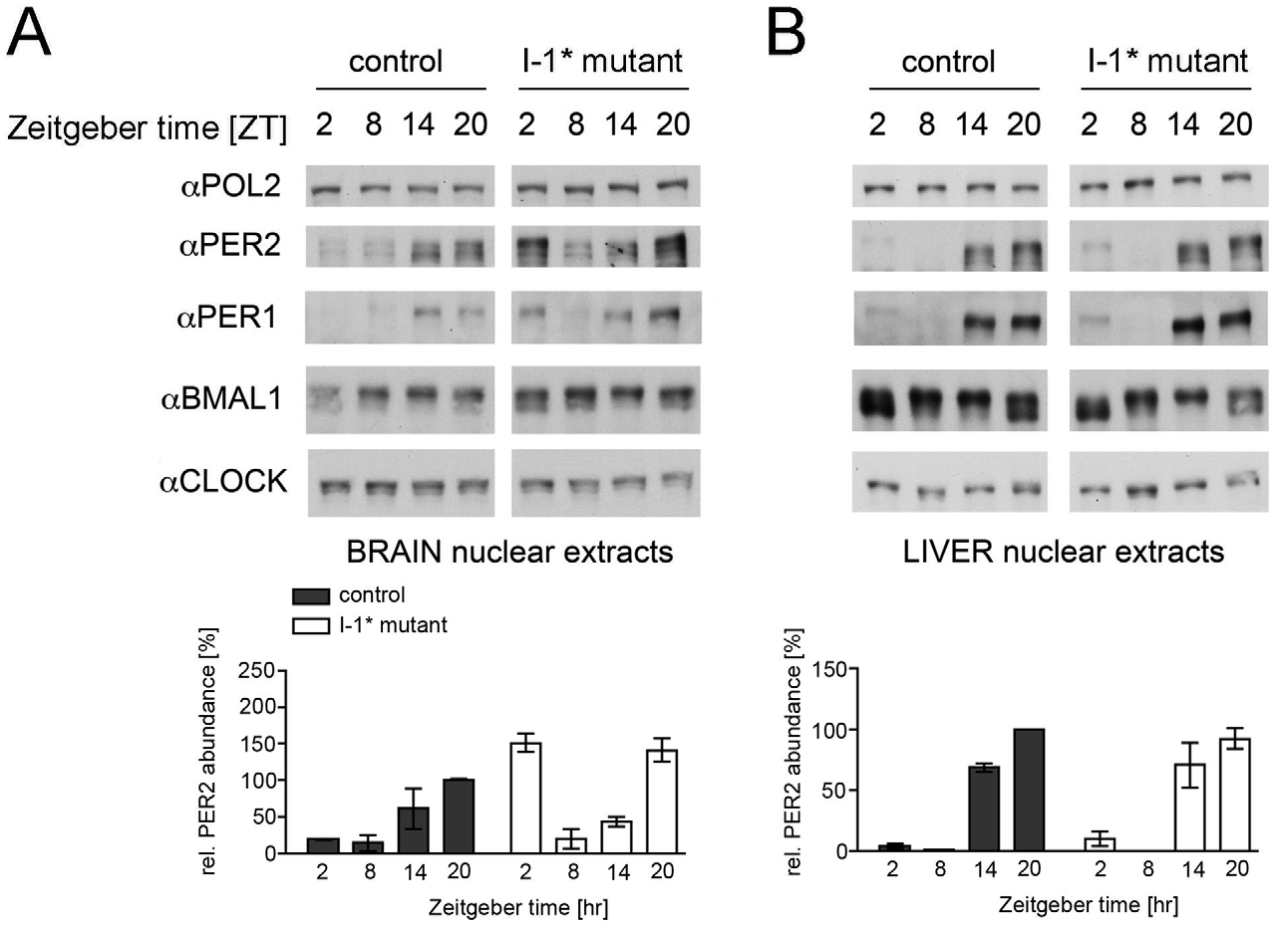
Protein expression in nuclear extracts from brain and liver. Nuclear extracts from coronal brain slices containing the SCN (A; pool of two animals) and liver (B) from control and I-1* mutant mice under on dox conditions were prepared at 6-h intervals from mice held in a 12-h light/12-h dark cycle (LD 12∶12 h cycle). The protein accumulation patterns were analyzed by Western blot analysis using the indicated antibodies. The accumulation of RNA polymerase II (antiPOL2) is shown as loading control. Below the quantification of PER2 levels in nuclear extracts from brain and liver is shown (n = 4 animals; t-test control vs. mutant at ZT2 and ZT20, p<0.05 for brain, p>0.05 for liver).

A previous study suggests that PP1 targets the clock component PER2 *in vitro* [33]. To test whether modulating PP1 activity affects clock protein expression or localization *in vivo*, we analyzed the clock proteins in nuclear extracts prepared from coronal brain slices containing the SCN or from liver tissue (as not I-1* expressing tissue). Control animals and I-1* mutant mice maintained in a LD 12∶12 h cycle under on dox conditions were sacrificed every six hours. We observed altered accumulation patterns of PER1 and PER2 in brain nuclear extracts from I-1* mutant mice compared to nuclear extracts from control animals (Figure 4A). I-1* mutant animals displayed higher levels of nuclear PER1 and PER2 at ZT2 and ZT20. To a lower extent this appears also to be the case for BMAL1. By contrast, CLOCK nuclear accumulation was similar in both, extracts from control and I-1* mutant mice. Moreover, expression patterns of PER1, PER2, BMAL1 and CLOCK were comparable in liver extracts from the same animals (Figure 4B) indicating that the observed effect was due to the brain-specific expression of I-1*.

Altered PER protein accumulation observed in brain nuclear extracts might be due to changes in the overall amount of PER proteins or changes in their intracellular localization. To get insights on this, we analyzed PER2 expression in the SCN of I-1* mice by immunohistochemistry. Immunohistochemical stainings comparing the expression of PER2 in the SCN of control and I-1* mice revealed PER2 to be rhythmically expressed in the SCN of both genotypes, with the higher amount of antiPER2-immunoreactive nuclei present at ZT14 and ZT20 (Figure S4A). No apparent differences regarding phase and magnitude of PER2 expression were found between control and I-1* mutant mice under induced conditions. However, the analysis of immunohistochemical stainings at ZT2 at a higher magnification indicated slight differences in the subcellular distribution of the antiPER2 signal in control and mutant animals (Figure S4B). In SCN sections from I-1* mutant mice, elevated levels of nuclear PER2 were detected compared to control animals (62.6% in I-1* mutant animals and 48.9% in control animals, Figure S4C).

### I-1* expression alters the subcellular localization of PER2 *in vitro*

**Figure 5:**
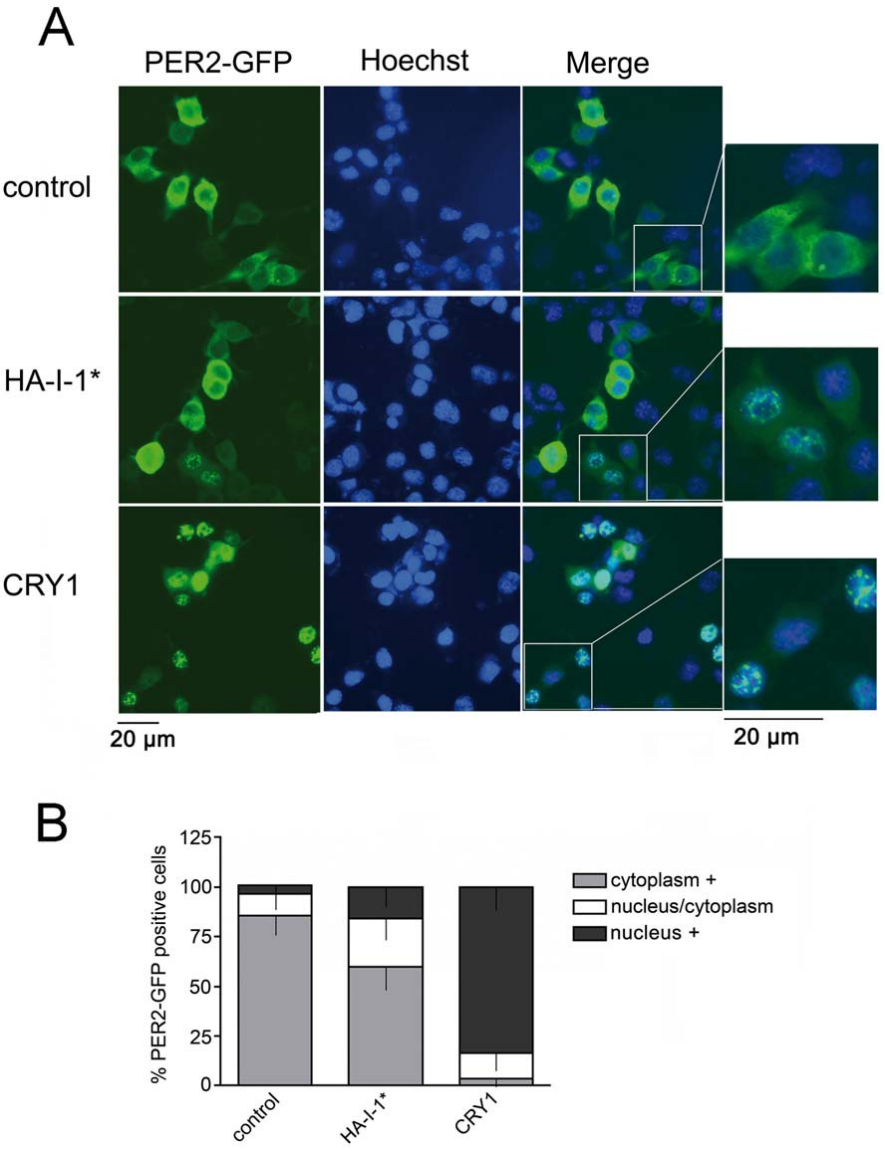
Inhibition of PP1 in cells modulates PER2 subcellular localization. (A) NG108-15 cells were transfected with PER2-GFP together with either empty expression vector (control, upper panels), expression vector for HA-tagged I-1* (HA-I-1*; middle panels), or CRY1 (lower panels). Representative fluorescent microscopic images are shown. From left: EGFP fluorescence (green), Hoechst-stained nuclear fluorescence (blue) and a merge of these images. Scale bar: 20 µm. (B) Quantitative distribution of the subcellular expression of PER2-GFP in NG108-15 cells. Cells were transfected with PER2-GFP together with either empty expression plasmid, expression vector for HA-I-1*, or expression vector coding for CRY1. Cells were assessed for predominantly nuclear, nuclear and cytoplasmic, or predominantly cytoplasmic PER2-GFP expression. Relative distribution was determined blinded in three independent experiments. The distribution was evaluated in at least 80 GFP-positive cells for each experiment. Data are represented as mean + SD.

In order to confirm our observations in SCN tissue we investigated the effects of I-1* expression on the localization of the PER2 protein in cultured cells. To this end we analyzed the expression and subcellular localization of a GFP-tagged version of PER2 in NG108-15 neuroblastoma cells, since this cell line is of neuronal and glial origin. GFP-PER2 was expressed either with empty expression vector or expression vectors for I-1* or CRY1. In NG 108-15 cells, exogenously expressed PER2-GFP was found predominantly in the cytoplasm, although some cells expressed PER2-GFP also in the nucleus (Figure 5A, 5B). Co-expression of the clock component CRY1 promoted nuclear localization of the PER2 protein (3.4%±6.2 nuclear localization in control cells versus 83.5%±11.4 in CRY1-expressing cells; p<0.001; Figure 5). This is in agreement with previous studies [13], [47]. Compared to control cells, significantly more PER2 localized to the nucleus when I-1* was co-expressed with PER2-GFP in NG 108-15 cells (3.4%±6.2 nuclear localization in control cells versus 16.0%±9.5 in HA-I-1*-expressing cells; p<0.01; Figure 5). Both the fraction of cells expressing PER2-GFP in the nucleus, and in nucleus/cytoplasm increased (Figure 5B). The same tendency was observed, when NIPP1* was co-expressed with PER2 (Figure S5A, S5B). The nuclear accumulation of PER2 was not due to a general effect of the inhibitor expression on GFP localization, as the subcellular localization of GFP-tagged beta-Catenin was not influenced in these cells (Figure S5C).

In conclusion it appears that I-1* expression and thereby PP1 inhibition in the brain does not have a major effect on the total amount of PER2 in neurons. However, our experiments indicate that PP1 may be involved in the regulation of the subcellular localization of the PER2 protein.

## Discussion

Post-translational modifications of clock components such as ubiquitination, acetylation and phosphorylation have critical functions in the mammalian circadian clock [21]. A basic transcriptional-translational feedback loop alone would operate only in a range of a few hours [48]. Post-translational regulation is an elegant way to add delays of several hours between activation and repression of transcription and consequently to adjust the period of the circadian clock to the 24-hour day. By regulating clock protein stability, activity, and/or subcellular localization, protein phosphorylation plays a crucial role in the maintenance of the 24-hour rhythm. Several clock components are subject to phosphorylation (reviewed in [21]). Among the mammalian clock proteins, phosphorylation of the PER proteins appears to have a special importance for the dynamics of the circadian cycle.

Kinases of the casein kinase (CK) 1 and 2 family have been shown to determine the period of the circadian system by targeting the PER proteins [24], [25], [49], [50]. Furthermore, specific phosphorylated residues in the PER proteins affect the period length of the oscillator. The FASPS mutation of hPER2 (S662G) results in PER2 being hypophosphorylated by CK1 *in vitro* [26]. Interestingly, mice carrying this mutation display a shorter period length in DD [27]. By contrast, the mutation of the FASPS site of hPER2 to a phosphomimicking aspartate residue leads to a significantly longer period. This result highlights that phosphorylation events at specific residues are dynamic but are highly balanced to maintain circadian cycles of about 24 hours. Reversibility is achieved by the interplay of protein kinases and phosphatases.

Studies in several organisms suggested functions of protein serine/threonine phosphatases in regulating circadian rhythmicity [28], [29], [30], [31]. Therefore, we were interested to see, whether PP1 has important functions in the mammalian clock mechanism. We provide evidence that this enzyme influences the dynamics of the mammalian circadian timing system. Down-regulation of the catalytic subunits of PP1 by RNAi and of PP1 activity by over-expression of a constitutively active inhibitor I-1* lengthened the period of luciferase rhythms in cells (Figure 1). Furthermore, mice expressing constitutively active PP1 inhibitors in the brain displayed behavioral rhythms with a longer period length (Figure 2C) than control mice, suggesting a function of PP1 in regulating the period of the circadian oscillator *in vivo*. This phenotype is similar to the phenotype observed in fruit flies having reduced PP1 activity [30]. Flies over-expressing NIPP1 in clock neurons exhibited a significantly lengthened circadian period. Interestingly, these flies also showed high amplitude light-induced resetting. The same phenotype was observed in I-1* as well as NIPP1* mutant mice (Figure 3). Hence, the role of PP1 in controlling clock parameters may be evolutionarily conserved at least between *Drosophila* and mammals.

PP1 is an ubiquitous protein serine/threonine phosphatase that has been implicated in various biological processes [34]. By the specific interaction of the PP1 catalytic subunits with various regulatory and targeting proteins, activity and substrate specificity are highly regulated. In a previous study, PP1 was shown to coimmunoprecipitate with, dephosphorylate and stabilize the PER2 protein in HEK293 cells [33]. In our study, we found only a minor effect of PP1 on the stability of PER2 in the brain and of overexpressed full-length PER2 in NG 108-15 neuroblastoma cells (Figure S4A, 5, S6). In particular, expression of PP1 inhibitors (HA-I-1* or HA-NIPP1*) did not affect the half-life of V5-tagged full-length PER2 in NG 108-15 cells treated with cycloheximide (Figure S6). This discrepancy may be due to tissue-specific differences in PP1 action on PER2 that may involve different regulatory components. However, comparing protein expression patterns in brain nuclear extracts, we found differences in the nuclear accumulation of PER1 and PER2 between control and I-1* mutant mice (Figure 4). Moreover, a larger fraction of PER2-GFP was located in the nucleus of neuroblastoma cells expressing I-1* or NIPP1* compared to control cells (Figure 5, S5). From these results, we conclude that PP1 influences the subcellular localization of PER2 by tipping the PER2 cytoplasmic to nuclear ratio towards the cytoplasmic fraction. Taken together, our and previous studies suggest PER2 as a molecular target of PP1. Timing of nuclear entry and the dynamics of nuclear accumulation of the components of the repressor complexes containing PER2 influence the period of the circadian oscillator [22], [24]. Hence, we can speculate that PP1 impacts on the period partially via the PER2 protein. However, a slight effect of PP1 inhibition was observed for BMAL1, indicating that PP1 may not act on PER protein accumulation only, but may also affect BMAL1 levels possibly via epigenetic modifications on the transcriptional machinery [43].

In addition to affecting oscillator function under constant conditions, we show that PP1 is involved in the mechanisms in the SCN that mediate the response of the oscillator to entraining light input (Figure 3). Entrainment of the clock employs several signal transduction cascades that depend on protein phosphorylation (for review see [41]). A recent study shows that the pleckstrin homology domain leucine-rich repeat protein phosphatase 1 (PHLPP1), which counteracts Akt, protein kinase C (PKC), and ERK1/2 signaling, impacts on resetting by modifying the period length after a light stimulus [51]. This further highlights the importance of balanced activities between protein kinases and phosphatases in the entrainment mechanism of the circadian clock system.

The signaling pathways mediating light input to the clock involve phospho-CREB induced *Per1* and *Per2* expression in the SCN [14], [15]. Our data indicate that PP1 does not significantly affect the immediate early signaling cascades in the SCN that ultimately lead to *Per* gene induction. *Per1* and *Per2* induction after light pulses at CT14 or CT22 was similar in control and I-1* mice (Figure S3). Hence, PP1 may not influence resetting of the clock via CRE-mediated mechanisms inducing *Per* gene expression in the SCN. Instead, PP1 may influence resetting of the molecular oscillator in the SCN by acting on the phosphorylation status of the PER proteins. Similarly, PKC has been shown to influence phase shifting properties of the circadian clock by affecting stability and subcellular distribution of PER2 in response to light input [23]. In addition to the function of PP1 in the resetting mechanisms of the SCN, this phosphatase appears also to be involved in retinal transmission of light to the SCN specifically at early subjective night [52].

Altogether, our experiments suggest that PP1 is a post-translational regulator of the mammalian circadian clock *in vivo*. This phosphatase modulates the period of the circadian oscillator and serves as a negative regulator in the light-induced resetting mechanisms in the SCN. We hypothesize that PP1 fulfills these functions (at least in part) by influencing the dynamics of PER2 nuclear accumulation.

## Materials and Methods

### Plasmids

The synthetic open reading frame (ORF) coding for constitutively active Inhibitor 1 (I-1*; [37]) and the coding region for the fragment of Nuclear Inhibitor of PP1 (NIPP1*; [43]) were cloned in frame into HA-pSCT1 (expressing the protein with a double N-terminal HA-tag). PER2-EGFP and pSTC-CRY1 expression vectors are described [53]. The ORF coding for a N-terminally truncated form of beta-Catenin was cloned into pEGFPC3 and then subcloned into the pSTC expression vector.

### Virus production

RNAi constructs targeting PP1 catalytic subunits (PPP1CA/CB/CC) and non-silencing RNAi as control were purchased from Open Biosystems. The corresponding oligo-IDs are indicated in Table S1. For lentivirus production HEK293T cells (Thermo Scientific #HCL4517) were seeded in T25 flasks and transfected with 2.5 µg psPAX, 1.5 µg pMD2G and 3.5 µg of the respective lentiviral expression plasmid (RNAi constructs) using the *CalPhos™* Mammalian Transfection *Kit* (BD-Biosciences) according to the manufacturer's protocol.

Virus-containing supernatants were filtered and U-2 OS (human, American Type Culture Collection [ATCC] # HTB-96) reporter cells stably expressing firefly luciferase from a 0.9-kb *Bmal1* promoter fragment were transduced with 1 ml of virus filtrate plus 8 ng/µL protamine sulfate in 35 mm dishes. After 1 d, medium was exchanged to puromycin-containing (10 µg/mL) medium for selection.

### Bioluminescence recording of transduced U-2 OS cells

3 days after transduction reporter cells were synchronized with dexamethasone (1 µM) for 30 min. After washing with 1×PBS, cells were cultured in phenol-red-free DMEM medium containing 10% fetal calf serum, antibiotics, and 250 µM D-luciferin (Biothema). Light emission was recorded at 37°C in a LumiCycle photomultiplier advice (Actimetrics) for 4–6 d. Raw data were detrended by dividing the 24-h-running average. Periods were estimated by fitting the cosine wave function as described [24].

### Determination of knock-down efficiency by Quantitative PCR

For testing knockdown efficiencies of the respective RNAi constructs, cells were harvested immediately after bioluminescence recording. Total RNA was prepared using RNeasy Mini Kit (Qiagen, Hilden) and then reversely transcribed to cDNA using Superscript RNase H- (Invitrogen). Quantitative PCR was performed with SYBRGreen fluorescence assays using human QuantiTect Primer Assays (Qiagen) and analyzed by ABI PRISM 7000 detection system (Applied Biosystems). The transcript levels for each clock gene were normalized to *hGapdh* and evaluated according to the 2-deltadeltaCt method [54].

### Real-time bioluminescence monitoring of transfected NIH 3T3 cells

Proliferating NIH 3T3 mouse fibroblasts [62] cultured on 35-mm dishes in DMEM High Glucose (Sigma-Aldrich) supplemented with 100 U/ml penicillin/streptomycin (Amimed) and 10% fetal calf serum (Amimed) were transfected with *Bmal1 ext. luciferase* reporter vector [55], pCMV SEAP for normalization, and the indicated expression vectors using linear polyethylenimine (Polysciences Europe) according to the manufacturer's instructions. 48 hours after transfection, cells were synchronized by addition of DMEM containing 100 nM dexamethasone [56]. After 20 min, the medium was changed to phenol red-free DMEM supplemented with 5% fetal calf serum and 0.1 mM luciferine. Bioluminescence was recorded in real-time using a LumiCycle apparatus (Actimetrics). Differences in transfection efficiencies between different cultures were corrected for by normalization to the secreted alkaline phosphatase activity (SEAP activity; Roche Applied Science) measured in a culture medium sample taken before synchronization of the cells. Bioluminescence recordings were analyzed with LumiCycle analysis software (Actimetrics).

### Animals

Animal care and handling was performed in accordance with the guidelines of the Schweizer Tierschutzgesetz (TSchG, SR455) and the declaration of Helsinki. The protocol was approved by the state veterinarian of to the Canton of Fribourg (permit FR 11/10). tetO-I-1* mice [38] or tetO-NIPP1* mice [43] were crossed with mice expressing the reverse tetracycline controlled transactivator (rtTA2) under the Ca^2+^/CaM-dependent protein kinase IIalpha (*CaMKIIalpha*) promoter [39] to obtain double transgenic mice. As controls, littermates carrying no transgene or one allele of either transgene were used. For doxycycline treatment, mice were fed doxycycline containing normal chow at 6 mg per 1 g food for at least 8 days. Adult male mice (3–8 months old) were used for behavioral experiments.

### Protein phosphatase assay

PP1 protein phosphatase assays were performed using whole cell SCN extracts as previously described [43]. Tissue containing the SCN was dissected out of a 1 mm thick coronal brain slice using a mouse brain slicer (Zivic instruments).

### Locomotor activity and circadian phenotype analysis

Mice housing and handling was performed as described [42]. Animals were tested for their circadian phenotype without doxycycline treatment (off dox), during doxycyline treatment (on dox), and after doxycycline treatment (on/off dox). For on/off dox conditions, doxycycline-containing chow was replaced by normal chow and animals were analyzed for circadian behavior after a waiting period of at least 10 days. For LD – DD transitions, lights were turned off at the end of the light phase and not turned on again the next morning. We used the Clocklab software (Actimetrics) for wheel running data acquisition and analysis. Activity records are double-plotted so that each day cycle's activity is plotted both to the right and below that of the previous cycle. The free-running period length was determined on 10 consecutive days of stable free-running activity in constant darkness by χ^2^-periodogram analysis. For light-induced phase shifts, we used the Aschoff type I protocol as described [42]. Animals were kept in constant darkness for at least 21 days before and after light administration [15 min bright white light (400 Lux) at circadian times (CT) CT10, CT14 and CT22]. The phase shift was determined by fitting regression lines through at least 7 consecutive onsets of activity before the light pulse and at least 7 consecutive onsets of activity after the light pulse. The two days following the light pulse were not considered for the evaluation of the phase shift. The difference between the two regression lines on the first day after the light pulse determined the phase shift.

### Protein extraction and Western blot analysis

Cell nuclei from mouse brain and liver were purified with the method described in [57]. To obtain sufficient brain material, 2 mm thick coronal brain slices containing the SCN were cut using a mouse brain slicer (Zivic instruments). The material from two mouse brain slices containing two SCN pairs was pooled and homogenized for one experiment. Nuclear extracts were prepared using the NUN procedure [58]. SDS-PAGE and immunoblot analysis were performed according to standard protocols. Antibodies used were rabbit PER1, PER2, BMAL1, CLOCK [59], RNA POLII (Bethyl). Quantification was performed using the Quantity One 1-D analysis software (Biorad). Relative PER2 abundance was calculated by defining the control PER2 level at ZT20 of each experiment as 100% (n = 2 individual experiments with a total of 4 animals).

### *In situ* hybridization

Animals (on dox) were exposed to a 15 min light pulse (400 lux) at either CT14 or CT22. 45 min after the end of the light pulse mice were sacrificed. Control animals were sacrificed without prior light exposure. Specimen preparation, ^35^S-UTP labeled riboprobe synthesis and hybridization steps were performed as described earlier [60]. The *in situ* hybridization probes were for *mPer1* and *mPer2* as described [61]. Quantification was performed by densitometric analysis of autoradiograph films (Amersham Hyperfilm MP) using the Quantity One 1-D analysis software (Biorad). Data from the SCN were normalized by comparison with the signal intensities in an equal area of the lateral hypothalamus. For each time point and genotype, 2–4 animals were used and 6 sections per SCN were analyzed. Relative mRNA abundance values were calculated by defining the wild-type control value of each experiment as 1.

### Immunohistochemistry

Tissue fixation in 4% PFA, dehydration, embedding and sectioning was performed as described for the *in situ* hybridization [60]. Following removal of embedding medium, 7 µm sections were rehydrated and boiled in 0.01 M Na-Citrate pH 6/ 0.05% Tween-20 for 10 min to unmask hidden antigen epitopes. After two washing steps in 1× PBS pH 7.4, the specimens were incubated in 3% H_2_O_2_ to inhibit endogenous peroxidases. Sections were blocked for 1 hour at room temperature in 10% serum, incubated overnight with the primary antibody at 4°C in humidified chamber and processed for immunohistochemical detection by using the Vectastain ABC kit (Vector laboratories) and diaminobenzidine tetrahydrochloride (DAB) as substrate (Sigma-Aldrich). The antibody used was rabbit antiPER2 (Alpha Diagnostic).

### Imaging and evaluation of GFP expression

NG108-15 neuroblastoma×glioma hybrid cells [63] were grown on poly-L-lysine coated coverslips in DMEM High Glucose (Sigma-Aldrich) supplemented with 10% FCS (Amimed) and 100 U/ml penicillin/streptomycin (Amimed) and were transfected with constructs coding for the indicated proteins. Transfection was performed using linear polyethylenimine (Polysciences Europe) according to the manufacturer's instructions. 24 hours after transfection, cells were fixed by incubation with 4% PFA and coverslips were mounted on glass slides. The cells expressing GFP-tagged proteins were analyzed and assigned to three different categories according to their predominantly nuclear, nuclear and cytoplasmic, or predominantly cytoplasmic expression. An examiner unconscious of the condition each image belonged to did the evaluation.

### Cycloheximide experiment

NG108-15 neuroblastoma×glioma hybrid cells [63] were grown on 10-cm dishes in DMEM High Glucose (Sigma-Aldrich) supplemented with 10% FCS (Amimed) and 100 U/ml penicillin/streptomycin (Amimed) and were transfected with equal amounts of pSCT1 Per2-V5 [55], and either empty vector, or vectors coding for HA-I-1* and HA-NIPP1* using linear polyethylenimine (Polysciences Europe) according to the manufacturer's instructions. After having reached confluency, cells were split on 5-cm dishes. After 48 hours, cells were treated with 25 µg/ml cycloheximide (Sigma) for 0, 1, 2, or 4 hours. Cells were washed twice with 1× PBS and resuspended in NDB buffer [100 mM KCl, 0.2 mM EDTA, 20% Glycerol, 20 mM Hepes pH 7.6, 2 mM DTT] and lysed with an equal volume of NUN buffer [600 mM NaCl, 2 M Urea, 2% NP-40, 50 mM Hepes pH 7.6] supplemented with protease and phosphatase inhibitors. Samples were analyzed by immunoblotting. Antibodies used were anti-V5 (Sigma) and anti-Actin (Sigma).

### Statistical analysis

Significant differences were determined using Prism 4 software (GraphPad). To assess the effect of genotype and treatment on period length in mice, a 2-way ANOVA analysis with repeated measures on the treatment factor was performed comparing control and mutant animals under off, on and on/off dox conditions. This was followed by Bonferroni post-tests comparing genotype within each level of treatment since there is a scientific justification not to be interested in the comparison of off dox control and on/off mutant, for example. Additionally, data sets were analyzed using paired t-tests. Other data were analyzed performing either unpaired t-test (to compare two groups) or a 1-way Anova with Bonferroni post-test (to compare several groups), as indicated in the figure legends. Values were considered significantly different with p<0.05 (*), p<0.01 (**), or p<0.001 (***).

## Supporting Information

Figure S1 **Controls of knock-down experiments.** (A) Knock-down efficiency of transduced RNAi constructs in U-2 OS cells. Expression levels of the indicated PP1 subunit (PPP1CA, PPP1CB, or PPP1CC) in cells transduced with the non-silencing construct were set to 1. (B) Average period length of *Bmal1 luc* oscillations in U-2 OS cells not transduced (medium) or transduced with a construct containing scrambled RNAi (non-silencing control) or antisense constructs against the catalytic subunits of PP1, respectively. Period length was determined in three independent experiments (with n = 2–3 cultures). For PPP1CB and PPP1CC two different antisense constructs were used. *** p<0.001, * p<0.05 indicating significance (1-way Anova). The oligo-ID's for the corresponding antisense constructs are indicated in table S1.
(TIF)

Figure S2 NIPP1* expression in the brain has minor effects on the circadian free-running period length. Average period length of control mice and NIPP1* mutants in constant darkness under off dox, on dox and on/off dox conditions. Data are presented as mean ± SEM.
(TIF)

Figure S3 **Light inducibility of** ***Per1*** **and** ***Per2*** is similar in control and I-1* mutant mice. (A) Induction of *Per1* expression in the SCN by a 15-min light pulse applied at CT14 as revealed by *in situ* hybridization. (B) Induction of *Per2* expression by a 15-min light pulse applied at CT14. (C) Induction of *Per1* expression by a 15-min light pulse applied at CT22. (D) Induction of *Per2* expression by a 15-min light pulse applied at CT22. Light treated and control animals were sacrificed at CT15 or CT23, respectively. The left panels show representative micrographs. The location within the SCN of each section was determined by Hoechst dye staining (blue). The *in situ* hybridization signal is given in yellow. The quantifications show means ± SEM of normalized optical densities. All experiments were performed with animals under doxycycline treatment (n = 2–4). Scale bar 200 µm.
(TIF)

Figure S4 PER2 expression in the SCN of control and I-1* mutant mice. (A) Immunohistochemical analysis of PER2 in the SCN of control and I-1* mutant mice under on dox conditions. Animals maintained in a LD 12∶12 h cycle were sacrificed every 6 hours. Representative micrographs are shown. The location of each section was determined by Hoechst dye staining. Scale bar 200 µm. (B) Immunohistochemical analysis of PER2 in the SCN at ZT2 at a higher magnification (×40). The top panels show the aPER2 staining, the middle panels the Hoechst dye staining and the lower panels represent the merge of the two stainings. Scale bar 20 µm. (C) Quantitative representation of subcellular localization of PER2 in the SCN at ZT2 in control and I-1* mutant mice. The quantification shows the percentage of cells showing nuclear (N), nuclear and cytoplasmic (N/C) and cytoplasmic PER2 staining. The quantification includes data from 5 SCN sections (approximately 80 nuclei/ section) for each genotype.
(TIF)

Figure S5 HA-NIPP1* expression alters the subcellular localization of PER2-GFP in NG108-15 cells. (A) Representative micrographs of cells transfected with expression vectors for PER2-GFP and HA-NIPP1*. Shown is the GFP fluorescence (green, left panel), the Hoechst-stain fluorescence (blue, middle panel) and the merge of the two images (right panel). (B) Quantitative representation of the subcellular localization of PER2-GFP expression in NG-108-15 cells. (C) Quantitative representation of the subcellular localization of beta-Catenin-GFP expression. NG108-15 cells were transfected with an expression vector for beta-Catenin-GFP together with either empty pSCT1 expression vector, pSCT1-HA-I-1*, or pSCT1-HA-NIPP1*. GFP positive cells were scored for predominantly nuclear, nuclear and cytoplasmic and predominantly cytoplasmic expression. The relative distribution was determined blinded in three independent experiments (out of 80 GFP-positive cells each). Data are represented as mean + SD.
(TIF)

Figure S6 **Cycloheximide treatment.** (A) NG-108-15 cells transiently expressing Per2-V5 either alone or together with HA-I-1* or HA-NIPP1* were treated for up to 4 hours with 25 µg/ml cycloheximide. Samples were analyzed by immunoblotting using the indicated antibodies. (B) Two individual experiments were quantified using the Quantity One 1-D analysis software (Biorad). For each time point, the amount of PER2-V5 was normalized to actin. For each experiment, the normalized amount of PER2-V5 at time point 0 was set to 100%.
(TIF)

Table S1 **Antisense constructs.**
(DOC)

Table S2 Period length of control and I-1* mutant mice.
(DOC)

## References

[1] Bass J, Takahashi JS (2010). Circadian integration of metabolism and energetics.. Science.

[2] Ko CH, Takahashi JS (2006). Molecular components of the mammalian circadian clock.. Hum Mol Genet.

[3] Bunger MK, Wilsbacher LD, Moran SM, Clendenin C, Radcliffe LA (2000). Mop3 is an essential component of the master circadian pacemaker in mammals.. Cell.

[4] Hogenesch JB, Gu YZ, Jain S, Bradfield CA (1998). The basic-helix-loop-helix-PAS orphan MOP3 forms transcriptionally active complexes with circadian and hypoxia factors.. Proc Natl Acad Sci U S A.

[5] DeBruyne JP, Weaver DR, Reppert SM (2007). CLOCK and NPAS2 have overlapping roles in the suprachiasmatic circadian clock.. Nat Neurosci.

[6] King DP, Zhao Y, Sangoram AM, Wilsbacher LD, Tanaka M (1997). Positional cloning of the mouse circadian clock gene.. Cell.

[7] Reick M, Garcia JA, Dudley C, McKnight SL (2001). NPAS2: an analog of clock operative in the mammalian forebrain.. Science.

[8] Zheng B, Albrecht U, Kaasik K, Sage M, Lu W (2001). Nonredundant roles of the mPer1 and mPer2 genes in the mammalian circadian clock.. Cell.

[9] Zheng B, Larkin DW, Albrecht U, Sun ZS, Sage M (1999). The mPer2 gene encodes a functional component of the mammalian circadian clock.. Nature.

[10] van der Horst GT, Muijtjens M, Kobayashi K, Takano R, Kanno S (1999). Mammalian Cry1 and Cry2 are essential for maintenance of circadian rhythms.. Nature.

[11] Gekakis N, Staknis D, Nguyen HB, Davis FC, Wilsbacher LD (1998). Role of the CLOCK protein in the mammalian circadian mechanism.. Science.

[12] Griffin EA, Staknis D, Weitz CJ (1999). Light-independent role of CRY1 and CRY2 in the mammalian circadian clock.. Science.

[13] Kume K, Zylka MJ, Sriram S, Shearman LP, Weaver DR (1999). mCRY1 and mCRY2 are essential components of the negative limb of the circadian clock feedback loop.. Cell.

[14] Motzkus D, Maronde E, Grunenberg U, Lee CC, Forssmann W (2000). The human PER1 gene is transcriptionally regulated by multiple signaling pathways.. FEBS Lett.

[15] Travnickova-Bendova Z, Cermakian N, Reppert SM, Sassone-Corsi P (2002). Bimodal regulation of mPeriod promoters by CREB-dependent signaling and CLOCK/BMAL1 activity.. Proc Natl Acad Sci U S A.

[16] Akiyama M, Minami Y, Nakajima T, Moriya T, Shibata S (2001). Calcium and pituitary adenylate cyclase-activating polypeptide induced expression of circadian clock gene mPer1 in the mouse cerebellar granule cell culture.. J Neurochem.

[17] Albrecht U, Zheng B, Larkin D, Sun ZS, Lee CC (2001). MPer1 and mper2 are essential for normal resetting of the circadian clock.. J Biol Rhythms.

[18] Tischkau SA, Mitchell JW, Tyan SH, Buchanan GF, Gillette MU (2003). Ca2+/cAMP response element-binding protein (CREB)-dependent activation of Per1 is required for light-induced signaling in the suprachiasmatic nucleus circadian clock.. J Biol Chem.

[19] Wakamatsu H, Takahashi S, Moriya T, Inouye ST, Okamura H (2001). Additive effect of mPer1 and mPer2 antisense oligonucleotides on light-induced phase shift.. Neuroreport.

[20] Gallego M, Virshup DM (2007). Post-translational modifications regulate the ticking of the circadian clock.. Nat Rev Mol Cell Biol.

[21] Vanselow JT, Kramer A, Albrecht U (2010). Posttranslational regulation of circadian clocks.. The circadian clock.

[22] Vanselow K, Vanselow JT, Westermark PO, Reischl S, Maier B (2006). Differential effects of PER2 phosphorylation: molecular basis for the human familial advanced sleep phase syndrome (FASPS).. Genes Dev.

[23] Jakubcakova V, Oster H, Tamanini F, Cadenas C, Leitges M (2007). Light entrainment of the mammalian circadian clock by a PRKCA-dependent posttranslational mechanism.. Neuron.

[24] Maier B, Wendt S, Vanselow JT, Wallach T, Reischl S (2009). A large-scale functional RNAi screen reveals a role for CK2 in the mammalian circadian clock.. Genes Dev.

[25] Meng QJ, Logunova L, Maywood ES, Gallego M, Lebiecki J (2008). Setting clock speed in mammals: the CK1 epsilon tau mutation in mice accelerates circadian pacemakers by selectively destabilizing PERIOD proteins.. Neuron.

[26] Toh KL, Jones CR, He Y, Eide EJ, Hinz WA (2001). An hPer2 phosphorylation site mutation in familial advanced sleep phase syndrome.. Science.

[27] Xu Y, Toh KL, Jones CR, Shin JY, Fu YH (2007). Modeling of a human circadian mutation yields insights into clock regulation by PER2.. Cell.

[28] Comolli J, Taylor W, Rehman J, Hastings JW (1996). Inhibitors of serine/threonine phosphoprotein phosphatases alter circadian properties in Gonyaulax polyedra.. Plant Physiol.

[29] Yang Y, He Q, Cheng P, Wrage P, Yarden O (2004). Distinct roles for PP1 and PP2A in the Neurospora circadian clock.. Genes Dev.

[30] Fang Y, Sathyanarayanan S, Sehgal A (2007). Post-translational regulation of the Drosophila circadian clock requires protein phosphatase 1 (PP1).. Genes Dev.

[31] Sathyanarayanan S, Zheng X, Xiao R, Sehgal A (2004). Posttranslational regulation of Drosophila PERIOD protein by protein phosphatase 2A.. Cell.

[32] Eide EJ, Woolf MF, Kang H, Woolf P, Hurst W (2005). Control of mammalian circadian rhythm by CKIepsilon-regulated proteasome-mediated PER2 degradation.. Mol Cell Biol.

[33] Gallego M, Kang H, Virshup DM (2006). Protein phosphatase 1 regulates the stability of the circadian protein PER2.. Biochem J.

[34] Ceulemans H, Bollen M (2004). Functional diversity of protein phosphatase-1, a cellular economizer and reset button.. Physiol Rev.

[35] Cohen PT (2002). Protein phosphatase 1–targeted in many directions.. J Cell Sci.

[36] Foulkes JG, Strada SJ, Henderson PJ, Cohen P (1983). A kinetic analysis of the effects of inhibitor-1 and inhibitor-2 on the activity of protein phosphatase-1.. Eur J Biochem.

[37] Alberts AS, Montminy M, Shenolikar S, Feramisco JR (1994). Expression of a peptide inhibitor of protein phosphatase 1 increases phosphorylation and activity of CREB in NIH 3T3 fibroblasts.. Mol Cell Biol.

[38] Genoux D, Haditsch U, Knobloch M, Michalon A, Storm D (2002). Protein phosphatase 1 is a molecular constraint on learning and memory.. Nature.

[39] Michalon A, Koshibu K, Baumgartel K, Spirig DH, Mansuy IM (2005). Inducible and neuron-specific gene expression in the adult mouse brain with the rtTA2S-M2 system.. Genesis.

[40] Alvarez-Saavedra M, Antoun G, Yanagiya A, Oliva-Hernandez R, Cornejo-Palma D (2010). miRNA-132 orchestrates chromatin remodeling and translational control of the circadian clock.. Hum Mol Genet.

[41] Hirota T, Fukada Y (2004). Resetting mechanism of central and peripheral circadian clocks in mammals.. Zoolog Sci.

[42] Jud C, Schmutz I, Hampp G, Oster H, Albrecht U (2005). A guideline for analyzing circadian wheel-running behavior in rodents under different lighting conditions.. Biol Proced Online.

[43] Koshibu K, Graff J, Beullens M, Heitz FD, Berchtold D (2009). Protein phosphatase 1 regulates the histone code for long-term memory.. J Neurosci.

[44] Gau D, Lemberger T, von Gall C, Kretz O, Le Minh N (2002). Phosphorylation of CREB Ser142 regulates light-induced phase shifts of the circadian clock.. Neuron.

[45] Ginty DD, Kornhauser JM, Thompson MA, Bading H, Mayo KE (1993). Regulation of CREB phosphorylation in the suprachiasmatic nucleus by light and a circadian clock.. Science.

[46] Reppert SM, Weaver DR (2002). Coordination of circadian timing in mammals.. Nature.

[47] Yagita K, Tamanini F, Yasuda M, Hoeijmakers JH, van der Horst GT (2002). Nucleocytoplasmic shuttling and mCRY-dependent inhibition of ubiquitylation of the mPER2 clock protein.. EMBO J.

[48] Ripperger JA, Brown SA, Albrecht U (2010). Transcriptional regulation of circadian clocks.. The circadian clock.

[49] Camacho F, Cilio M, Guo Y, Virshup DM, Patel K (2001). Human casein kinase Idelta phosphorylation of human circadian clock proteins period 1 and 2.. FEBS Lett.

[50] Vielhaber E, Eide E, Rivers A, Gao ZH, Virshup DM (2000). Nuclear entry of the circadian regulator mPER1 is controlled by mammalian casein kinase I epsilon.. Mol Cell Biol.

[51] Masubuchi S, Gao T, O'Neill A, Eckel-Mahan K, Newton AC (2010). Protein phosphatase PHLPP1 controls the light-induced resetting of the circadian clock.. Proc Natl Acad Sci U S A.

[52] Yan L, Bobula JM, Svenningsson P, Greengard P, Silver R (2006). DARPP-32 involvement in the photic pathway of the circadian system.. J Neurosci.

[53] Albrecht U, Bordon A, Schmutz I, Ripperger J (2007). The multiple facets of Per2.. Cold Spring Harb Symp Quant Biol.

[54] Livak KJ, Schmittgen TD (2001). Analysis of relative gene expression data using real-time quantitative PCR and the 2(−Delta Delta C(T)) Method.. Methods.

[55] Schmutz I, Ripperger J, Baeriswyl S, Albrecht U (2010). The mammalian clock component PERIOD2 coordinates circadian output by interaction with nuclear receptors.. Genes Dev.

[56] Balsalobre A, Brown SA, Marcacci L, Tronche F, Kellendonk C (2000). Resetting of circadian time in peripheral tissues by glucocorticoid signaling.. Science.

[57] Ripperger JA, Shearman LP, Reppert SM, Schibler U (2000). CLOCK, an essential pacemaker component, controls expression of the circadian transcription factor DBP.. Genes Dev.

[58] Lavery DJ, Schibler U (1993). Circadian transcription of the cholesterol 7 alpha hydroxylase gene may involve the liver-enriched bZIP protein DBP.. Genes Dev.

[59] Preitner N, Damiola F, Lopez-Molina L, Zakany J, Duboule D (2002). The orphan nuclear receptor REV-ERBalpha controls circadian transcription within the positive limb of the mammalian circadian oscillator.. Cell.

[60] Oster H, Werner C, Magnone MC, Mayser H, Feil R (2003). cGMP-dependent protein kinase II modulates mPer1 and mPer2 gene induction and influences phase shifts of the circadian clock.. Curr Biol.

[61] Albrecht U, Sun ZS, Eichele G, Lee CC (1997). A differential response of two putative mammalian circadian regulators, mper1 and mper2, to light.. Cell.

[62] Jainchill JL, Aaronson SA, Todaro GJ (1969). Murine sarkoma and leukemia viruses: assay using clonal lines of contact-inhibited mouse cells.. J Virol.

[63] Klee WA, Nierenberg M (1974). A neuroblastoma times glioma hybrid cell line with morphine receptors.. Proc Natl Acad Sci USA.

