# Supplementary figures and images for: Protein Phosphatase 1 (PP1) Is a Post-Translational Regulator of the Mammalian Circadian Clock
Source: PLoS One. 2011 Jun 21;6(6):e21325. doi: 10.1371/journal.pone.0021325 (PMC3119686; doi:10.1371/journal.pone.0021325)

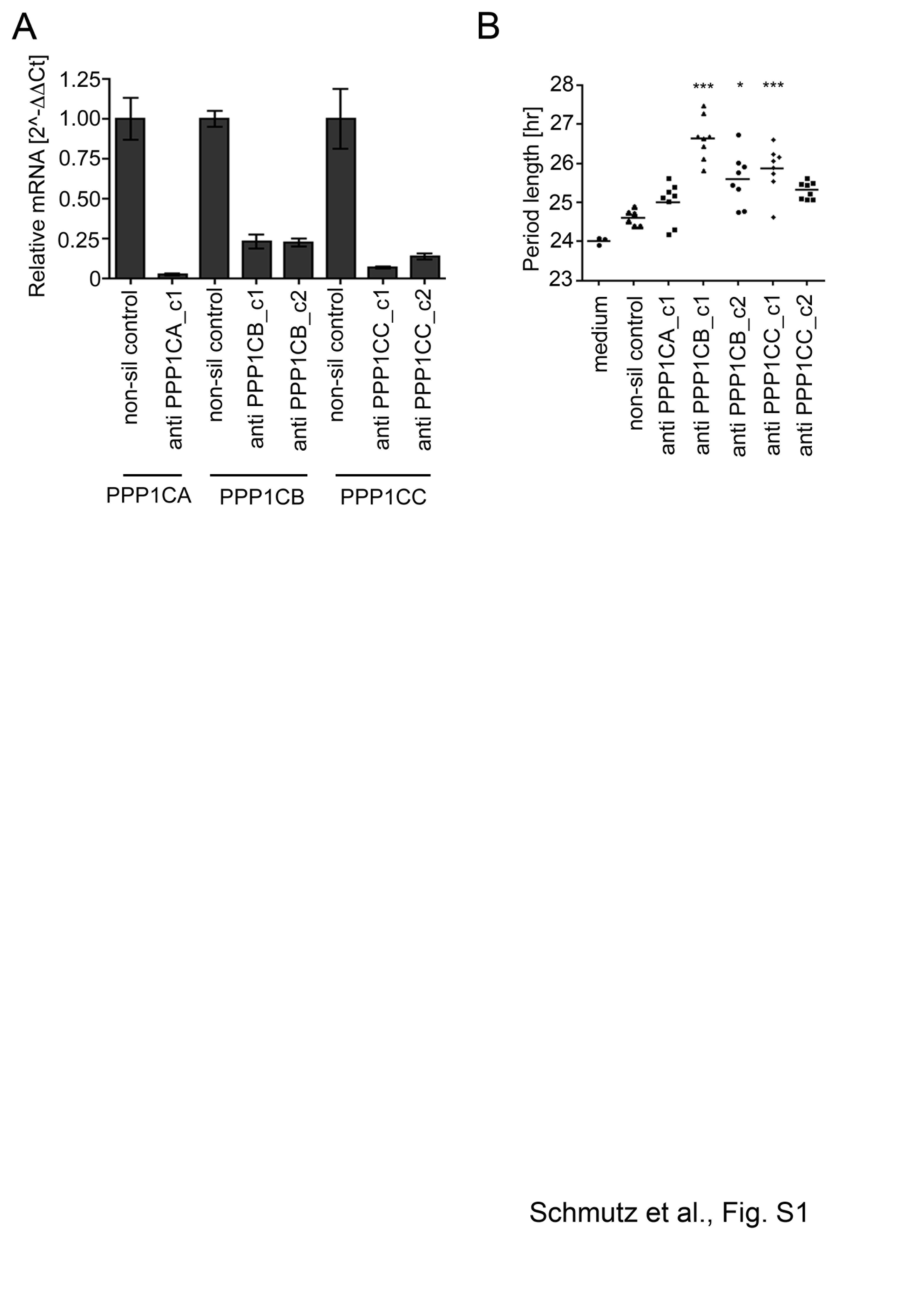

Supplement: Figure S1 — Controls of knock-down experiments. (A) Knock-down efficiency of transduced RNAi constructs in U-2 OS cells. Expression levels of the indicated PP1 subunit (PPP1CA, PPP1CB, or PPP1CC) in cells transduced with the non-silencing construct were set to 1. (B) Average period length of Bmal1 luc oscillations in U-2 OS cells not transduced (medium) or transduced with a construct containing scrambled RNAi (non-silencing control) or antisense constructs against the catalytic subunits of PP1, respectively. Period length was determined in three independent experiments (with n = 2–3 cultures). For PPP1CB and PPP1CC two different antisense constructs were used. *** p<0.001, * p<0.05 indicating significance (1-way Anova). The oligo-ID's for the corresponding antisense constructs are indicated in table S1. (TIF) [file pone.0021325.s001.tif]

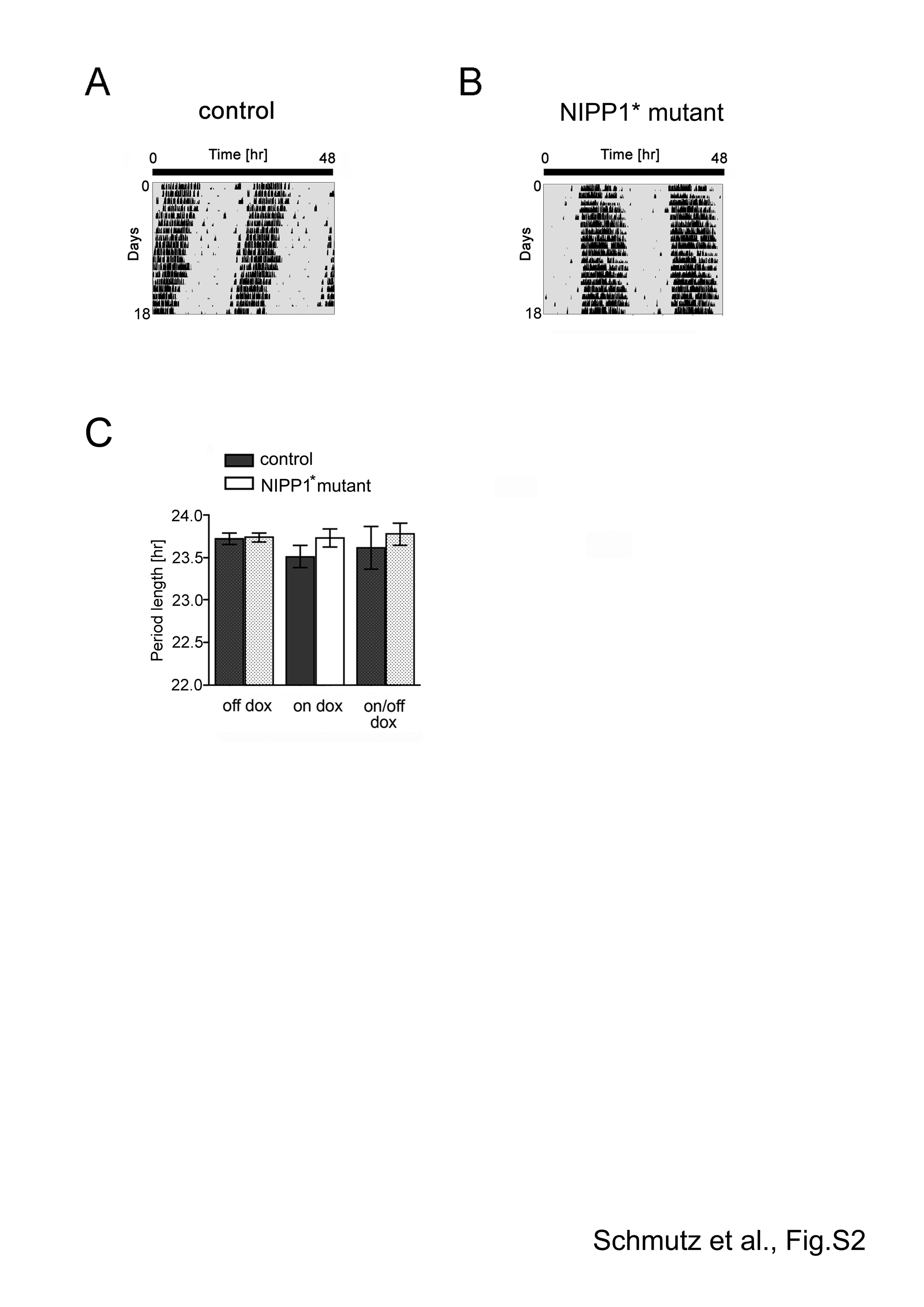

Supplement: Figure S2 — NIPP1* expression in the brain has minor effects on the circadian free-running period length. Average period length of control mice and NIPP1* mutants in constant darkness under off dox, on dox and on/off dox conditions. Data are presented as mean ± SEM. (TIF) [file pone.0021325.s002.tif]

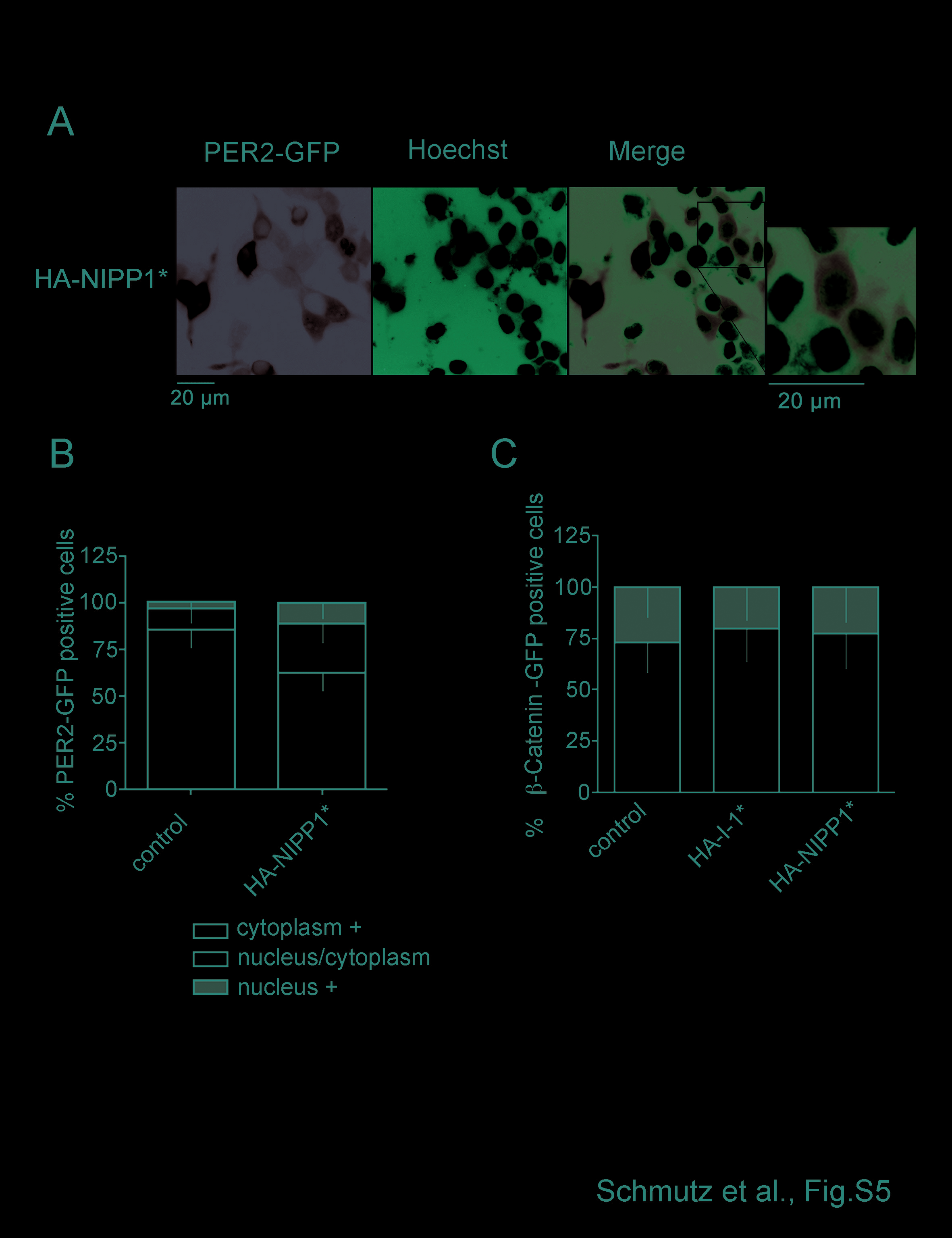

Supplement: Figure S5 — HA-NIPP1* expression alters the subcellular localization of PER2-GFP in NG108-15 cells. (A) Representative micrographs of cells transfected with expression vectors for PER2-GFP and HA-NIPP1*. Shown is the GFP fluorescence (green, left panel), the Hoechst-stain fluorescence (blue, middle panel) and the merge of the two images (right panel). (B) Quantitative representation of the subcellular localization of PER2-GFP expression in NG-108-15 cells. (C) Quantitative representation of the subcellular localization of beta-Catenin-GFP expression. NG108-15 cells were transfected with an expression vector for beta-Catenin-GFP together with either empty pSCT1 expression vector, pSCT1-HA-I-1*, or pSCT1-HA-NIPP1*. GFP positive cells were scored for predominantly nuclear, nuclear and cytoplasmic and predominantly cytoplasmic expression. The relative distribution was determined blinded in three independent experiments (out of 80 GFP-positive cells each). Data are represented as mean + SD. (TIF) [file pone.0021325.s005.tif]
