# Supplementary material for: Protein Phosphatase 1 (PP1) Is a Post-Translational Regulator of the Mammalian Circadian Clock
Source: PLoS One. 2011 Jun 21;6(6):e21325. doi: 10.1371/journal.pone.0021325 (PMC3119686; doi:10.1371/journal.pone.0021325)
Supplement: Table S1 — Antisense constructs. (DOC) [file pone.0021325.s007.doc]

| **Antisense construct** | **Oligo-ID (Open Biosystems)** |
| --- | --- |
| anti PPP1CA_c1 | V2LHS_262414 |
| anti PPP1CB_c1 | V2LHS_223243 |
| anti PPP1CB_c2 | V2LHS_220925 |
| anti PPP1CC_c1 | V2LHS_170309 |
| anti PPP1CC_c2 | V2LHS_170312 |
| non-sil control | Non-silencing GIPZ Lentiviral shRNAmir control RHS4346 |

Table S1: Antisense constructs
