# Supplementary material for: Protein Phosphatase 1 (PP1) Is a Post-Translational Regulator of the Mammalian Circadian Clock
Source: PLoS One. 2011 Jun 21;6(6):e21325. doi: 10.1371/journal.pone.0021325 (PMC3119686; doi:10.1371/journal.pone.0021325)
Supplement: Table S2 — Period length of control and I-1* mutant mice. (DOC) [file pone.0021325.s008.doc]

| **Comparison** | **p value (paired t-test)** |
| --- | --- |
| Control off dox versus control on dox | p < 0.0001 |
| Control on/off dox versus control on dox | p = 0.0035 |
| Control off dox versus control on/off dox | p = 0.0002 |
| I-1* mutant off dox versus I-1* mutant on dox | p = 0.0033 |
| I-1* mutant on/off dox versus I-1* mutant on dox | p = 0.0706 |
| I-1* mutant off dox versus I-1* mutant on/off dox | p = 0.0648 |

Table S2: Period length of control and I-1* mutant mice (p-values of paired t-test; control n=14; mutant n=9)
